# Psychometric Proprieties of a Mobile Application to Measure the Craniovertebral Angle a Validation and Reliability Study

**DOI:** 10.3390/ijerph17186521

**Published:** 2020-09-08

**Authors:** Tomas Gallego-Izquierdo, Enrique Arroba-Díaz, Gema García-Ascoz, María del Alba Val-Cano, Daniel Pecos-Martin, Roberto Cano-de-la-Cuerda

**Affiliations:** 1 Physiotherapy and Pain Research Group. Physiotherapy Department, Faculty of Medicine and Health Sciences, University of Alcalá, Alcalá de Henares, Plaza de San Diego, s/n, 28801 Alcalá de Henares, Madrid, Spain; tomas.gallego@uah.es (T.G.-I.); enrique.fisio94@gmail.com (E.A.-D.); gema.garcia.ascoz@gmail.com (G.G.-A.); albavalcano94@gmail.com (M.d.A.V.-C.); 2 Physiotherapy, Occupational Therapy, Rehabilitation and Physical Medicine Department, Universidad Rey Juan Carlos, 28933 Móstoles, Madrid, Spain; roberto.cano@urjc.es

**Keywords:** head, mobile applications, posture, sensitivity, specificity, validation study

## Abstract

The aim of this study was to assess the psychometric properties of the mobile application forward head posture in terms of validity, inter- and intra-rater reliability, minimum detectable change, sensitivity, and specificity to measure craniovertebral angle. In total, 44 subjects (mean age 23.30 ± 4.44 years) were evaluated in the standing position with markers on the tragus and cutaneous prominence of seventh cervical vertebra (C7). We had two experienced and trained physiotherapists assess cervical posture using the mobile application forward head posture and photogrammetry. Intraclass correlation coefficients were used to determine validity and reliability. A contingency table was made to determine sensitivity and specificity. Intra-rater reliability of the mobile application forward head posture had an intraclass correlation coefficient of 0.88. The inter-rater reliability generated an intraclass correlation coefficient of 0.83 to 0.89. Criterion validity data were above 0.82. The minimum detectable change was 4.96° for intra-rater and 5.52° for inter-rater reliability. The smartphone application exhibited 94.4% sensitivity and 84.6% specificity. The smartphone application forward head posture is a valid and reliable tool to measure craniovertebral angle in a standing position and, therefore, could be a useful assessment tool in clinical practice.

## 1. Introduction

Posture can be defined as the ability to maintain a correct relationship between the different body segments within the limits of stability in relation to environment and activities [1]. An adequate posture maintains a correct body scheme with minimal effort, allowing for balance in the musculoskeletal system [2]. Appropriate craniocervical posture occurs when the head is slightly anterior to the cervical spine so that the earlobe and the shoulder can be joined by a vertical line [3]. In physiotherapy, many assessment tests are performed in a standing position because it does not require excessive effort [4].

Cervical pain is a frequent cause of medical consultation. It is estimated that more than half of the world’s population suffers from cervicalgia at some time in their life [5]. Cervical pain is related to posture and poor body alignment at that position [6,7]. Forward head posture (FHP) is one of the most common postural disorders of the cervical spine [8]. In this posture, there is extension of the upper cervical vertebrae and flexion of the lower ones [9]. This poor alignment is associated with the increased use of electronic devices, among other causes, such as long periods of study in school-aged children or sedentary office work in adults [10]. 

Disorders at the cervical level, such as FHP, can cause multiple problems, including neck pain and disability. Therefore, analyzing cervical posture and detecting postural disorders are important to determine their correlation with pain or dysfunction and to determine if there are changes after therapeutic interventions in craniocervical positioning [11,12].

Classically, cervical posture has been evaluated by measuring three angles: The craniovertebral angle (CV), the cervical inclination angle, and the inclination angle of the head. The CV angle is the best indicator of FHP [6]. To determine this angle, two references are defined: One line that runs from the swallow of the ear to seventh cervical vertebra (C7) and another horizontal line parallel to the ground that passes only through the spinous apophysis of C7 [6]. An angle less than 50°–53° may indicate FHP [2,6,13]. Thereby, the smaller the CV angle, the greater the disability [14]. 

CV angle is the most reliable and valid indicator of FHP [15]. However, there is currently no standard method for its measurement. One of the most extensive methods involves the use of a photogrammetry system and the subsequent analysis of the photograph from a sagittal plane using postural evaluation software [6]. This method has many advantages, as it is relatively fast, the image and values obtained are easily preserved, and it is more precise and reliable than visual evaluation alone [15]. Therefore, photogrammetry is considered the ‘gold standard’ for assessing head position [16,17]. A camera and even mobile application [16] can be used for registration.

Mobile applications have become a good alternative to photogrammetry systems because they simplify the process by streamlining image acquisition and data analysis and reducing costs. This study aimed to examine the psychometric properties of the FHP mobile application (FHPapp) in terms of its validity, intra- and inter-rater reliability, minimum detectable change, sensitivity, and specificity in measuring the CV angle.

## 2. Materials and Methods

### 2.1. Participants 

A total of 44 voluntary subjects from the University of Alcalá (Alcalá de Henares, Spain) were recruited by the nonprobabilistic sampling of consecutive cases. Study subjects were between 18 and 65 years old with or without cervical pain, with no history of a whiplash injury or cervical spine arthrodesis, and did not suffer from tinnitus, vertigo, dizziness, or feelings of instability during the study. 

Every participant provided their age, weight (body mass index (BMI) was calculated), presence and neck pain intensity (quantified using the visual analogue scale (VAS)), regular practice of exercise, and hours of exercise per week. 

The data was anonymized and the ethical guidelines for clinical research in humans outlined in the Helsinki Declaration (and subsequent revisions) were followed [18]. This study was approved by the “Comité de Ética de Investigación y Experimentación Animal” (Ethical Research and Animal Experimentation Committee) of the Universidad de Alcalá (CEIM/HU/2018/33). All participants were provided with an information sheet and an informed consent sheet prior to the start of the measurements.

### 2.2. Procedure

#### FHPapp

FHPapp (Pyeongtaek, South Korea) has been available since 2016 and its objective is to determine the cervical posture by measuring the CV angle. It is an application that requires an Android version 4.0 or higher installed on a smartphone. It currently has 50,000–100,000 downloads.

This application allows users to measure the CV angle from a sideview photograph. The application also describes four phases of head positioning and provides an unvalidated questionnaire to establish the diagnosis of head anteriorization.

### 2.3. Measurement Procedure

In this study, the CV angle was evaluated with FHPapp on a Xiaomi^®^ MiA2 (Beijing, China) and with a Canon^®^ EDS100D camera (Tokyo, Japan). The images were subsequently analyzed using the free Kinovea^®^ software.

The following evaluation protocol was established: Two markers were placed on the ground separated by 1.5 m, where the camera or smartphone and the subject were located. Reference markers were placed on the spinous process of C7 and in the swallow of the subjects’ ears, which were identified through palpitation. The subject was placed on the floor mark, barefoot and standing, lateral to the evaluator. At this point, two evaluators took photographs in two sessions. The evaluators were experienced and trained physiotherapists.

In the first session, the first evaluator took a picture with a smartphone and then with a camera. Then, the second evaluator performed the same sequence. This was repeated for all participants. The measurement order of the subjects was random. The second measurement was performed one week later using the same protocol.

Once the images were obtained, the smartphone image was introduced in FHPapp to obtain the CV angle. The application provides the complement to the CV angle, thus making further calculation necessary. To determine the CV angle, it is necessary to subtract the angle provided by the application from 360°. The image obtained with the camera was uploaded onto an Asis^®^ laptop for analysis with the Kinovea software. This program has the advantage of directly providing the CV angle without the need for additional calculation.

Once the CV angle was obtained, all measures—the ones obtained in the analysis with Kinovea^®^ and the ones calculated with the FHPapp—were classified attending to the presence or not of FHP. The presence of FHP (FHP group) was determined by an angle value lower than 50°, and the absence of FHP (no-FHP group) if the angle value was greater than or equal to 50° [6,15].

### 2.4. Statistical Analysis 

The SPSS program version 25.0 (IBM, Chicago, IL, USA) for Windows was used to perform the statistical analyses. For descriptive variables and those corresponding to the CV angle, a descriptive frequency analysis was performed. From the rest of the variables, the mean was presented with its 95% confidence interval (CI) limits and standard deviation (SD).

The Interclass Correlation Coefficient (ICC) calculation was used with a 95% CI accompanied by the standard error of the mean (SEM) SEM = SD $\sqrt{1-\mathrm{ICC}}$ and its graphical representation in the Bland and Altman diagrams to study intra-observer reliability (facing the measurements made with application by an observer in the first and second session), inter-observer reliability (facing the measures of the first and second session with application of the different observers), and the criterion validity (facing measurements made with FHPapp and photogrammetry in the different sessions) [19]. These measurements were accompanied by the minimum detectable change (MDC) with the formula MDC = 1.96 $\sqrt{2\;\mathrm{SEM}}$ [20]. ICC values between 0.50 and 0.69 were considered “moderate,” from 0.70 to 0.89 “high,” and above 0.90 “excellent” [19].

For the study of the sensitivity and specificity of the application as evidence indicating the presence of FHP, a receiver operating characteristic (ROC) curve and a contingency table were used, using photogrammetry with Kinovea^®^ software as a gold standard [11,17]. Accuracy was assessed using the area under the curve (AUC). Values from 0.50 to 0.70 indicate low precision, 0.70 to 0.90 moderate precision, and higher than 0.90 high precision [21].

The sample size was calculated using the software “Tamaño de la muestra 1.1.” (developed by Pérez Medina et al.). Data were calculated using an intraclass correlation coefficient (ICC) of 0.83 [7], an alpha error of 0.05 and a precision level of 0.16. Using these values, the estimated sample size was 30 participants. 

## 3. Results

A total of 44 subjects were evaluated—21 women and 23 men—with a mean age of 23.30 ± 4.44 years (mean ± SD). A descriptive analysis of the demographic data is shown in Table 1.

**Figure 1:**
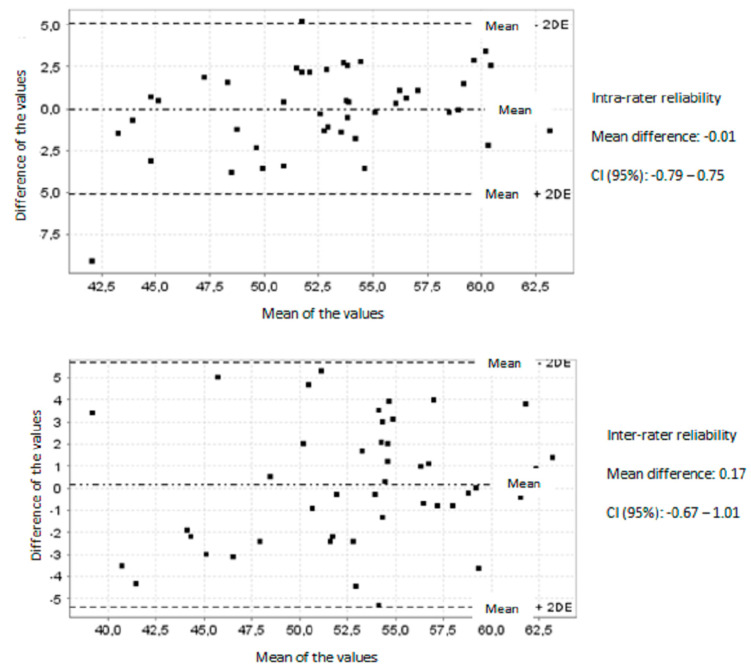
Bland and Altman chart for intra- and inter-observer reliability.

After performing the FHP application reliability analysis, a high value of intra-rater reliability was found with an intraclass correlation coefficient (ICC) of 0.88 for one of the observers. The corresponding Bland and Altman graph is shown in Figure 1. The inter-rater reliability was also high, with ICC values ranging from 0.83 to 0.89 and an average of 0.85. The Bland and Altman graph of inter-rater reliability with an ICC of 0.89 is shown in Figure 1. The SEM and MDC values are shown in Table 2 with the intra- and inter-rater reliability scores.

The ICC values obtained for the validity between FHPapp and photogrammetry were above 0.82, with a maximum value of 0.88 (CI: 0.82–0.91) (Table 3). These high values indicate that FHPapp is a valid tool to assess cervical posture.

**Figure 2:**
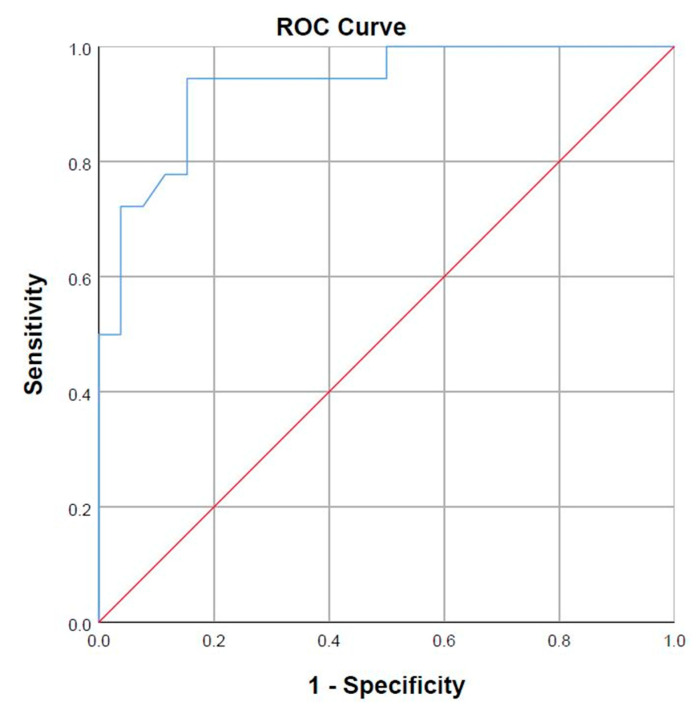
Sensitivity and specificity of FHPapp (COR (Characteristic Operating Receiver) curve).

The AUC was 0.93 (Figure 2). After adjusting the cutoff point to 0.333, the FHPapp demonstrated a sensitivity of 94.4% and specificity of 84.6% for the assessment of FHP (Table 4).

## 4. Discussion

The objective of this study was to evaluate the validity and intra- and inter-rater reliability of FHPapp. Our results indicated high values for both intra- and inter-rater reliability, and this mobile application also presented a high sensitivity and specificity with values of 94.4% and 84.6%, respectively.

FHPapp is a free mobile application that enables measurement of the CV angle for the analysis of cervical posture in the sagittal plane. In the present study, it was used to measure the CV angle. The protocol involved: (1) Preparation of the location of the camera and the subject; (2) palpation and marking anatomical reference points; (3) realization of the photographs. Our procedure was performed in a standing position based on studies from Shaghayeghfard et al. [6], which the measurement of the CV angle in a seated position was found to lead to classification of “without FHP” in subjects with alterations of said angle.

No software was used in the analysis of the anatomical references. An agreement was reached between evaluators to choose a specific point within the marker (most distal point of the marker attached to the subject’s body). According to Furlanetto et al. [15], using different reference points or evaluation software could affect the angle measurement, which would make it difficult to compare results.

The intra- and inter-rater reliability showed high ICC values in all measurements. These results are in line with other studies in the advanced head measurement. Ruivo et al. [2] found an ICC for inter-rater reliability of 0.83 to 0.88 using the postural software (PAS) and a cutoff point to determine FHP of 52°. Nam et al. [12] compared visual analysis against photogrammetry to obtain an ICC of 0.75 to 0.91 with a cutoff point of 54°. Salahzadeh et al. [15] obtained an ICC of 0.89 to 0.92 when comparing visual evaluation with the analysis of photographs using the Adobe Acrobat Software. When Guan et al. [16] studied the cervical region in different positions with a camera and analyzed the image with the ImageJ software, they obtained an ICC of 0.92 to 0.97. All studies used the same anatomical references as our study (C7 and swallow of the ear) and also waited a week between measurements.

There are many factors that can contribute to the variability in the measurements, such as the evaluators (intra-rater reliability), the patients (intra-subject reliability), and the methodology (instrument-reliability) [3]. The high reliability found in our study could be conditioned by the practice of experienced examiners, as suggested by Nam et al. [12]. However, since reliability was not evaluated among inexperienced examiners, this cannot be conclusively affirmed, as in the study by Cheung et al. [14]. However, despite the high levels of reliability, the reference point for the measurement of the CV angle represents a limitation, since it is subject-dependent and influenced by the palpation of spinous references, as indicated by Dunleavy et al. [10].

Our results related to criterion validity suggest that FHPapp represents an alternative to photogrammetry. In this study, we used an image obtained with a digital camera subsequently treated with the free Kinovea^®^ Software. Other authors have used three-dimensional movement systems. However, these can be expensive [22]. The main tools used in the clinic to assess CV angle are direct visual observation [3,12] and low-cost technologies such as mobile applications [7,16]. Therefore, the objective of the present study was to determine the psychometric properties of FHPapp for use in the clinical evaluation of the CV angle.

As an FHP reference point, this study used an angle less than 50°, although, according to other studies, an angle less than 53° could be considered as FHP [6,15]. The results obtained in our sample were determined by the cutoff point chosen, as indicated in the study by Bosso et al. [4]. If the reference value changed, the classification of the subjects would also change, as established in the article by Salahzadeh et al. [15].

In this study, an MDC of 4.96 was obtained for intra-rater reliability and 5.52 for inter-rater reliability. These values are similar to those obtained by Dunleavy et al. [10] in their evaluation of the reliability of a device called Optotrak by calculating the ICC. This device assesses the range of cervical movement and was compared with an inclinometer, generating an MDC that ranged between 2.5 and 6.5. Cheung et al. [14] obtained an MDC of 3.31. They evaluated the validity and reliability of the Electronic Head Posture Instrument for measuring cervical position in subjects with and without neck pain, also by calculating the ICC.

According to Gadotti et al. [3] and Nam et al. [12], visual evaluations do not confer sufficient validity or reliability. Therefore, they may not detect small differences over time or in response to an intervention. Photogrammetry and FHPapp do detect such changes, as indicated by the ability to make reproducible measurements over time.

Compared to traditional photogrammetry [16,17], FHPapp was highly sensitive and specific. A sensitivity of 94.4% and specificity of 84.6% reflected that the application is a useful tool for identifying the presence of FHP. When attempting to compare these values, the lack of literature concerning this matter became apparent. There are studies [2,12,15,16] that have analyzed the similarity between intra- and inter-rater measurements, but none have analyzed the sensitivity or specificity of the measuring instrument or software in question. Certain smartphone applications have been used to analyze cervical movement [23] and spine posture [24,25,26]. These studies also failed to provide sensitivity and specificity data on these applications. However, having an easy-to-use, free mobile application to accurately determine the CV angle in clinical practice justifies its use, even in the absence of relevant comparison.

This research presents several limitations. The measurements made in this study were subject-dependent. This can lead to measurement and selection biases if body markers or measuring devices are not placed properly or if the data are misinterpreted. In addition, these biases can affect measurements made with a camera or a phone application. The study participants were all adult-aged and, since neither children nor the elderly were included, the results cannot be extrapolated. Furthermore, the results are not applicable to people with traumatic cervical events or other exclusion criteria used in this study. The present study describes only the alignment of the static spine in a standing position. Therefore, the findings cannot be generalized to alignment during the performance of functional tasks, especially when the upper limbs are in motion or loaded.

## 5. Conclusions

The use of FHPapp might be suitable for clinical practice and facilitates the measurement of cervical posture due to it is simplicity to use, lack of cost, and high accuracy. 

## Figures and Tables

**Table 1:** Demographic characteristics and descriptive values.

Variable	Female *n* = 21	Male *n* = 23	Total *n* = 44
Age (Years)	22.62 (±4.37) [20.63–24.61]	23.91 (±4.52) [21.96–25.87]	23.30 (±4.44) [21.94–24.65]
Weight (kg)	66.15 (±16.02) [58.86-73.45]	75.31 (±10.76) [70.66–79.97]	70.94 (±14.14) [66.54–75.24]
Height (cm)	166.64 (±5.98) [163.91–169.36]	177.15 (±5.04) [174.97–179.33]	172.13 (±7.60) [169.82–174.44]
Body Mass Index (BMI) (kg/m^2^)	23.84 (±5.99) [21.11–26.57]	23.93 (±2.68) [22.77–25.09]	23.88 (±4.51) [22.51–25.26]
Cervical Pain	13 (61.90%)	9 (39.13%)	n = 22 (50%)
Visual Analog Scale (VAS) (cm) Only Cervical Pain	2.65 (±1.20) [1.92–3.38]	4.43 (±1.12) [3.57–5.30]	3.38 (±1.45) [2.73–4.02]
Physical Activity Practice	15 (71.42%)	21 (91.30%)	*n* = 36 (81.81%)
Physical Activity (h/week) Only Physical Activity	5.43 (±3.39) [3.55–7.31]	5.45 (±2.06) [4.51–6.39]	5.44 (±2.65) [4.54–6.34]

**Table 2:** Intra- and inter-observer reliability.

	Observer	Mean (SD)	ICC (95% CI)	SEM	MDC
Intra-Observer Reliability	1 2	52.90° (±5.52) 52.79° (±5.17)	0.85 (0.74–0.91) 0.88 (0.79–0.93)	2.13° 1.79°	5.90° 4.96°
	Session	Mean (SD)	ICC (95% CI)	SEM	MDC
Inter-Observer Reliability	1 2	52.87° (±5.89) 52.82° (±4.74)	0.89 (0.80–0.93) 0.88 (0.80–0.93)	1.95° 1.64°	5.40° 4.54°

SD: Standard deviation. ICC: Intraclass correlation coefficient. CI: Confidence interval. SEM: Standard error of the mean. MDC: Minimum detectable change.

**Table 3:** FHPapp criterion validity.

	ICC (95% CI)	SEM	MDC
FHP1 vs. KIN1	0.85 (0.78–0.90)	2.11°	5.84°
FHP2 vs. KIN2	0.88 (0.82–0.91)	1.83°	5.07°

FHP1: The first observation from both observers with FHPapp. FHP2: Addition of the second observation from both observers with FHPapp. KIN1: First observation from both observers with Kinovea. KIN2: Addition of the second observation from both observers with Kinovea. ICC: Intraclass correlation coefficient. CI: Confidence interval. SEM: Standard error of the mean. MDC: Minimum detectable change.

**Table 4:** Sensitivity and specificity (contingency table).

		FHP Application		Correct Percentage (%)
		No FHP	FHP	
Photometry	No FHP	22	4	84.6
	FHP	1	17	94.4

FHP: Forward head posture.
